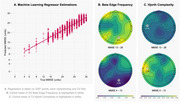# Cross‐Sectional Associations Between the Electroencephalogram and Cognitive Status: Toward Scalable Monitoring Solutions

**DOI:** 10.1002/alz70856_098538

**Published:** 2025-12-24

**Authors:** João Areias Saraiva, Martin Dyrba, Martin Becker, Ludwig Krause, Christoph Berger, Thomas Kirste, Stefan Teipel

**Affiliations:** ^1^ University of Rostock, Rostock, Mecklenburg‐Vorpommern, Germany; ^2^ German Center for Neurodegenerative Diseases (DNZE), Rostock, Mecklenburg‐Vorpommern, Germany; ^3^ University Medical Center of Rostock, Rostock, Mecklenburg‐Vorpommern, Germany; ^4^ German Center for Neurodegenerative Diseases (DZNE), Rostock, MV, Germany

## Abstract

**Background:**

Alzheimer's disease (AD) strains healthcare systems in an aging population, emphasizing the need for continuous cognitive decline monitoring and its early detection. The Mini‐Mental State Examination (MMSE) remains a widely used and cost‐effective diagnostic tool, with efforts underway to adapt it for digital home‐based assessments, enabling more frequent monitoring while minimizing patient burden and mobility. Similarly, electroencephalograms (EEG) have been investigated to monitor cognitive status in ambulatory settings. In this cross‐sectional study, we identified key EEG features reflecting the cognitive decline process and assessed their feasibility to estimate cognitive status using machine learning (ML).

**Method:**

An international and diverse cohort (France, Greece, Turkey, Argentina, Colombia) was gathered comprising *N* = 510 older adults (40‐98 years, 46% male). At the time of stationary EEG recording, subjects exhibited MMSE scores ranging from 30 (cognitively normal) to 4 (severe dementia). A Gradient Boosting ML regressor was developed to estimate their cognitive status based on their EEG spectrum, complexity, and connectivity, focusing on identifying features strongly associated with MMSE scores. The model estimations were evaluated in a leave‐one‐out cross‐validation procedure.

**Result:**

Key EEG features significantly correlated with MMSE scores included Hjorth Complexity in the left temporal lobe (r=0.58), alpha coherence between the left and right temporal lobes (r=0.48), and beta occipital edge frequency (r=0.42). Eighty combined EEG features were identified as predictors of cognitive status. Using these features, the ML regressor estimated cognitive status with an average error of 2.53 points in the MMSE scale (95% CI±5.36). The model demonstrated strong predictive performance, achieving an R^2^ value of 0.80 between estimated and actual MMSE scores.

**Conclusion:**

Specific EEG features, particularly those of temporal and occipital activity, can serve as reliable predictors of cognitive status. While cohort diversity enhanced the generalizability of these findings, more EEG recordings in the low MMSE range are needed to improve regression performance. Longitudinal studies are required to validate the tracking of intra‐subject EEG activity changes associated with cognitive decline. In the future, ML could automate periodic monitoring assessments of cognitive health based on EEG in its wearable and low‐resolution format, especially in regions with limited specialized staff and imaging technology.